# Nomogram for predicting pathologic complete response to neoadjuvant chemoradiotherapy in patients with esophageal squamous cell carcinoma

**DOI:** 10.1002/cam4.7075

**Published:** 2024-03-13

**Authors:** Guihong Liu, Tao Chen, Xin Zhang, Binbin Hu, Jiayun Yu

**Affiliations:** ^1^ Department of Radiotherapy, Cancer Center, State Key Laboratory of Biotherapy West China Hospital, Sichuan University Chengdu Sichuan China; ^2^ Department of Cardiology The First Affiliated Hospital of China Medical University Shenyang Liaoning China

**Keywords:** esophageal squamous cell carcinoma, neoadjuvant chemoradiotherapy, nomogram, pathologic complete response

## Abstract

**Purpose:**

A pathologic complete response (pCR) to neoadjuvant chemoradiotherapy (nCRT) is seen in up to 40% of the patients with esophageal squamous cell carcinoma (ESCC). No nomogram has been constructed for the prediction of pCR for patients whose primary chemotherapy was a taxane‐based regimen. The aim is to identify characteristics associated with a pCR through analyzing multiple pre‐ and post‐nCRT variables and to develop a nomogram for the prediction of pCR for these patients by integrating clinicopathological characteristics and hematological biomarkers.

**Materials and Methods:**

We analyzed 293 patients with ESCC who underwent nCRT followed by esophagectomy. Clinicopathological factors, hematological parameters before nCRT, and hematotoxicity during nCRT were collected. Univariate and multivariate logistic regression analyses were performed to identify predictive factors for pCR. A nomogram model was built and evaluated for both discrimination and calibration.

**Results:**

After surgery, 37.88% of the study patients achieved pCR. Six variables were included in the nomogram: sex, cN stage, chemotherapy regimen, duration of nCRT, pre‐nCRT neutrophil‐to‐lymphocyte ratio (NLR), and pre‐nCRT platelet‐to‐lymphocyte ratio (PLR). The nomogram indicated good accuracy and consistency in predicting pCR, with a C‐index of 0.743 (95% confidence interval: 0.686, 0.800) and a *p* value of 0.600 (>0.05) in the Hosmer–Lemeshow goodness‐of‐fit test.

**Conclusions:**

Female, earlier cN stage, duration of nCRT (< 62 days), chemotherapy regimen of taxane plus platinum, pre‐nCRT NLR (≥2.199), and pre‐nCRT PLR (≥99.302) were significantly associated with a higher pCR in ESCC patients whose primary chemotherapy was a taxane‐based regimen for nCRT. A nomogram was developed and internally validated, showing good accuracy and consistency.

## 1 INTRODUCTION

Esophageal cancer ranks the seventh most prevalent cancer globally and the sixth most prevalent cause of cancer‐induced mortality. Incidence rates are highest in southern and south‐central Asia, southern and eastern Africa, and northern Europe. The two major types of esophageal cancer are squamous cell carcinoma and adenocarcinoma. Squamous cell carcinoma is the predominant histological type of esophageal carcinoma in east Asia, including China where it accounts for over 90% of all cases of esophageal carcinoma.^1^ Surgery is the main treatment for esophageal cancer. Neoadjuvant chemoradiotherapy (nCRT) followed by surgery is the standard treatment for locoregional disease based on the success of the Dutch CROSS and NEOCRTEC5010 trials.^2^, ^3^

The purpose of nCRT is to downstage the primary tumor and regional lymph nodes and eliminate micrometastases. The optimal outcome includes a pathologic complete response (pCR), defined as the absence of malignant cells in the resected specimen.^4^ It is estimated that around 20%–40% of patients treated with nCRT would achieve a pCR, which has been demonstrated to be associated with improved recurrence‐free survival (RFS) and overall survival (OS).^5^, ^6^ A meta‐analysis confirmed that OS was comparable in patients with clinically complete response (cCR) after chemoradiotherapy undergoing active surveillance or standard esophagectomy.^7^ If pCR could be predicted with high accuracy, surgery could potentially be utilized as a salvage procedure instead of a planned procedure.^8^ Actually, current restaging tools, including positron emission tomography (PET), esophageal biopsy, and esophageal ultrasound, cannot reliably predict pCR after nCRT.^9^ Hence, researchers have been looking for new predictors for the efficacy of nCRT.

Although multiple clinical and biological parameters have been linked to a higher likelihood of achieving pCR, no single parameter can predict pCR with a probability of 40% or higher.^10^ The development of a model that predicts pCR with high accuracy could allow for the investigation of novel treatment strategies. A nomogram refers to a visual depiction of a mathematical equation that can be utilized as a means to predict the likelihood of clinical events. By incorporating multiple prognostic factors, nomograms have been shown to be more accurate than a single indicator. In view of high incidence of pCR and limited nomograms for predicting pCR for esophageal squamous cell carcinoma (ESCC), developing a nomogram that integrates multiple pre‐ and post‐nCRT parameters could be valuable in identifying patients with a high likelihood of pCR.^11^

The immune and inflammatory responses of the host are significant contributors to cancer development and treatment response. In various malignancies, the patient's prognosis has been associated with several hematological biomarkers of systemic immunoinflammation, including the neutrophil‐to‐lymphocyte ratio (NLR), platelet‐to‐lymphocyte ratio (PLR), and monocyte‐to‐lymphocyte ratio (MLR). Similarly, literature data uncovered that increased NLR, PLR, and MLR levels could be indicative of a poorer prognosis in ESCC patients who underwent surgical resection with or without neoadjuvant treatment.^12^ However, the relationship between inflammatory indicators and pCR remains to be explored. Pre‐nCRT MLR, not NLR and PLR, may predict pCR in 87 ESCC patients.^13^ A study enrolling 306 ESCC patients found that cases with pre‐nCRT NLR >2.1 showed a significantly lower pCR rate than those with lower value.^14^ Nevertheless, another study including 44 esophageal adenocarcinoma, 101 ESCC, and 4 undifferentiated carcinoma patients reported that baseline NLR was related to OS and RFS but not pCR, but PLR after surgery was associated with pCR.^15^ Surprisingly, the analysis of 311 patients with ESCC showed that pre‐nCRT PLR was significantly associated with pCR, but pre‐nCRT NLR and MLR were not associated with pCR.^16^ As mentioned above, the predictive capacity of pre‐CRT NLR, PLR, and MLR remains disputable.

One feature of the previously constructed prediction models was that chemotherapy regimens in nCRT were fluoropyrimidine plus platinum (PF) regimen or PF regimen accounted for the majority, while drugs represented by taxane are used more frequently than before in clinical practice due to their superior efficacy and ease of use.^14^, ^17^ Nevertheless, current studies have not constructed models for predicting pCR for such patients.

In this study, we aimed to identify potential indicators incorporating inflammatory factors that are associated with pCR after nCRT based on analyzing multiple pre‐ and post‐nCRT variables in a large cohort of patients with ESCC. Moreover, relevant parameters were selected by logistic regression method to construct a nomogram model integrating clinical features and immunity indices for predicting pCR. The performance of the resulting nomogram was internally validated by calculating the concordance statistic (c‐statistic) and the area under the receiver operating characteristic curve (AUROC). Calibration was evaluated by the Hosmer–Lemeshow goodness‐of‐fit test.

## 2 METHODS

### 2.1 Patients

ESCC patients who received nCRT followed by esophagectomy from the prospectively maintained database collecting the patients undergoing esophagectomy for all reasons at West China Hospital of Sichuan University from 2018 through 2021 were retrospectively analyzed.

The patients were included only if they had all of the following additional information: (1) baseline histologic confirmation, (2) pretreatment evaluation including physical examination, barium swallow test, chest/abdominal computed tomography (CT) with contrast, and (3) documentation of laboratory screens before and during nCRT, as well as prior to surgery. The Institutional Review Board at West China Hospital approved this analysis (2023‐1230).

### 2.2 Treatment and follow‐up

The chemotherapy regimen consisted of a fluoropyrimidine (intravenous or oral) or a taxane with or without a platinum compound.^18^, ^19^ The decision to perform an appropriate chemotherapy regimen and proper cycles of chemotherapy was taken by the treating oncologists based on their own clinical judgment according to the patient's condition. Patients received radiotherapy to a prescription dose of 40.0–50.4 Gy by either three‐dimensional conformal radiotherapy or intensity‐modulated radiotherapy. The gross tumor volume (GTV) included the primary tumor (GTVT) and potential metastatic lymph nodes (GTVN) based on enhanced CT scan. The clinical target volume (CTV) of the primary tumor provided a proximal and distal margin of 3 cm and a 5 mm radial margin around the GTVT to include the area of subclinical involvement. The CTV of potential metastatic lymph nodes is flared outward by 0.5 cm in each direction of GTVN. At the same time, natural anatomical boundaries such as the heart, lungs, bones, kidneys, and liver should also be avoided.^20^

All patients underwent minimally invasive McKeown esophagectomy.^21^ En bloc esophagectomy and complete two‐field lymph node dissection were performed as the standard procedure. Three‐field lymph node dissection was only done for patients with highly suspected cervical nodal disease. Cervical esophagogastrostomy was conducted using hand‐sewn double‐layer sutures.

Surgical specimens were reviewed by one experienced pathologist and a pCR was defined as no residual cancer cells in all layers of the resected esophagus and in the lymph nodes resected.^4^ Patients with any residual carcinoma on final pathology were considered non‐pCR to nCRT. After surgery, patients had check‐ups every 3 months for the first 2 years, every 6 months for the next 3 years, and then annually.

### 2.3 Data collection

Selection of parameters was partly based on previously published literature and partly on clinical experience. The parameters listed below were evaluated as potential predictors for pCR: sex, age at diagnosis, eastern cooperative oncology group performance status (ECOG PS) and numeric rating scale (NRS) scores, smoking, alcohol use, history of other diseases, body mass index (BMI), tumor length, cT stage, cN stage, cTNM stage, tumor location, radiotherapy dose, chemotherapy regimen, duration of nCRT, time interval between the end of nCRT and surgery, and hematological biomarkers.

Age was dichotomized into <60 versus ≥60. The pain assessment in patients employed the NRS scale, which ranges from 0 to 10. A score of 0 indicates no pain, while a score of 1–3 denotes mild pain, 4–6 indicates moderate pain, and 7–10 represents severe pain. NRS score was analyzed as 0 or greater than 0. Smoking and alcohol history contained three categories: no, yes, and yes but quit more than 1 year. Other medical histories include hypertension, diabetes, and hepatitis B virus. BMI before nCRT and before surgery was recorded, namely pre‐nCRT BMI and preoperative BMI, respectively. Pretreatment tumor length was defined as the maximum tumor length measured using a barium contrast agent. Staging was performed according to the American Joint Committee on Cancer Staging Manual, Eighth Edition.^22^ Radiotherapy dose was coded as ≤40Gy and >40Gy. The chemotherapy regimen simultaneously utilizing taxane and platinum agents (TP) throughout the entire nCRT period was considered as one type of chemotherapy regimen, while other cases were classified as separate. The regimen for TP refers to a taxane agent (paclitaxel 135 mg/m^2^, nab‐paclitaxel 260 mg/m^2^, or docetaxel 75 mg/m^2^) with a platinum agent (cisplatin 75 mg/m^2^, carboplatin area under the curve of 5 mg/mL/min, nedaplatin 80 mg/m^2^, or lobaplatin 50 mg/m^2^) on Day 1 every 3 weeks. The classification of interval between the end of nCRT and surgical resection was referenced from a study published in JAMA Surgery. The variable was divided into the subsequent categories: 0–42 days, 43–56 days, 57–70 days, 71–84 days, 85–98 days, and 99 or more days.^23^

Complete blood counts with differential, albumin, and globulin were recorded within a week before the initiation of nCRT. We calculated the albumin to globulin ratio (AGR) by dividing the albumin value with the globulin value. Similarly, the NLR, MLR, and PLR were calculated by dividing the absolute neutrophil count, absolute monocyte count, and platelet count by the absolute lymphocyte count, respectively. Toxicity was scored at each follow‐up according to the Common Terminology Criteria for Adverse Events (CTCAE), version 5.^24^

### 2.4 Statistical analysis

Continuous variables with a normal distribution were presented as mean ± standard deviation, and differences between groups were evaluated using the one‐way ANOVA test. Continuous variables with a skewed distribution were expressed as median with interquartile range, and differences between groups were compared with the Wilcoxon rank test. Chi‐squared tests were utilized to compare categorical data between groups. The optimal cutoff values of the duration of nCRT, AGR, NLR, NLR, and PLR for predicting pCR were determined using a receiver operating characteristic (ROC) curve.

To explore the relationship between parameters and pCR, a univariate logistic regression model was employed. The strength of the relationship was determined by odds ratios, along with 95% confidence intervals (CIs). The parameters with a *p* value of ≤0.1 in the univariate analysis were included in a multivariate logistic regression model by backward selection. IBM SPSS Statistics 25.0 software was used for statistical analysis.

A nomogram for predicting pCR was constructed using all parameters found to be statistically significant in the multivariate analysis. The performance of the nomogram was evaluated for both discrimination and calibration. Discrimination was assessed by calculating the c‐index (equal to the AUROC), which ranges from 0.5 (random prediction) to 1.0 (perfect discrimination), with higher values indicating better performance. Calibration was analyzed using a calibration curve to compare predicted and observed pCR rates. Calculations for developing the nomogram were conducted using the statistical software R. Statistical significance was defined as *p* < 0.05.

## 3 RESUTLS

### 3.1 General characteristics of the study participants

**FIGURE 1:**
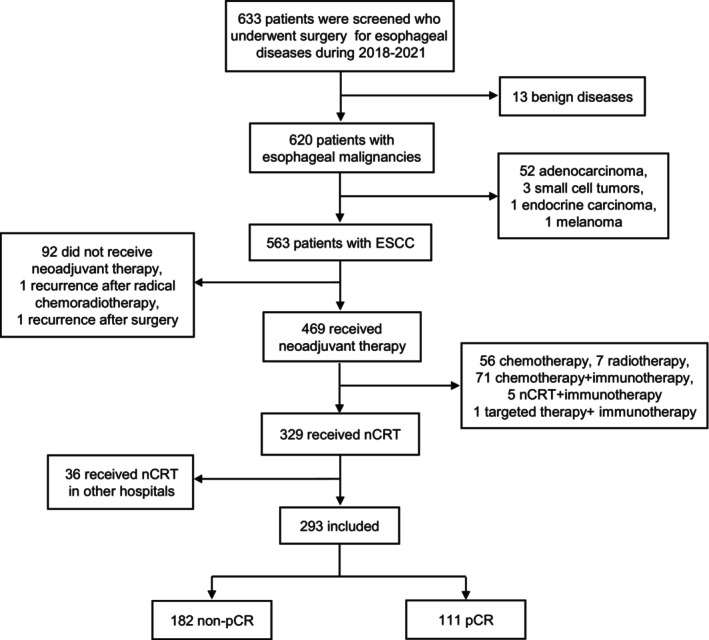
Flowchart of included patients. ESCC, esophageal squamous cell carcinoma; nCRT, neoadjuvant chemoradiotherapy; pCR, pathologic complete response.

A total of 633 patients were screened who underwent esophagectomy for any reason at our institution between 2018 and 2021. Among them, 563 patients were diagnosed with ESCC. Of these 563 ESCC patients, 329 were treated with nCRT followed by esophagectomy. After excluding 36 patients with incomplete data, the study included a total of 293 patients, of which 111 (37.88%) achieved pCR. No patient had a serious infection at the start of nCRT (Figure 1).

The majority of patients were male (81.9%) and a PS score of 0 to 1 (98.3%). More than half of the patients were over 60 years old and did not experience cancer‐related pain. The vast majority of individuals did not have comorbidities such as hypertension (81.6%), diabetes (94.9%), or hepatitis B (92.2%). The pretreatment mean tumor length was 5.231 cm. The majority of the tumors was located in the middle esophagus (63.5%). The study cohort was further characterized by an overrepresentation of cT3 tumors (66.9%) and cN1‐2 tumors (78.1%). The stages of the tumors were distributed as follows: 18.1% were Stage II, 55.3% were Stage III, and 26.6% were Stage IV. 58% of patients received a radiation dose greater than 40Gy. 87.7% utilized TP throughout the entire nCRT period. The chemotherapy regimen mentioned above was not applicable for 36 patients. Among them, 5 patients switched from the TP to T due to toxicity, 10 patients were treated only with T, 13 patients received PF regimen, 6 patients were given only F, and 2 patients received alternating TP and PF regimens. The largest proportion of individuals who underwent surgery after nCRT was between 57 and 70 days (30.4%), followed by 71–84 days (18.0%), 43–56 days (17.7%), and ≥ 99 days (15.0%). BMI at two time points and hematological indicators before nCRT were listed according to the data distribution. During nCRT, the majority of patients (71.3%) experienced leukopenia, and 52.2% of them experienced neutropenia. Grade ≥2 hemoglobinopenia was observed in only a minority of patients (12.3%), and 65.9% of patients did not develop thrombocytopenia (Tables 1 and 2).

**TABLE 1:** Patient characteristics and univariate analysis for categorical variables associated with pCR.

Categorical variable	pCR (%)	*p*‐value	Univariate analysis	
			OR (95% CI)	*p*‐value
Sex (*n*)				
Female (53)	30 (56.60%)		Reference	
Male (240)	81 (33.75%)	0.002	0.391 (0.213, 0.716)	0.002
Age, years (*n*)				
<60 (108)	45 (41.67%)		Reference	
≥60 (185)	66 (35.68%)	0.308	0.776 (0.477, 1.263)	0.308
PS (*n*)				
0 (216)	87 (40.28%)		Reference	
1 (72)	23 (31.94%)		0.696 (0.396, 1.225)	0.209
2 (5)	1 (20.00%)	0.319	0.371 (0.041, 3.373)	0.378
NRS (*n*)				
0 (189)	68 (35.98%)		Reference	
>0 (104)	43 (41.35%)	0.365	1.254 (0.768, 2.048)	0.365
Smoking history (*n*)				
No (114)	50 (43.86%)		Reference	
Yes (135)	44 (32.60%)		0.619 (0.369, 1.037)	0.068
Yes but quit (44)	17 (38.64%)	0.188	0.806 (0.396, 1.640)	0.552
Alcohol history (*n*)				
No (118)	53 (44.92%)		Reference	
Yes (147)	45 (30.61%)		0.541 (0.327, 0.896)	0.017
Yes but quit (28)	13 (46.43%)	0.036	1.063 (0.465, 2.429)	0.885
Hypertension (*n*)				
No (239)	90 (37.66%)		Reference	
Yes (54)	21 (38.89%)	0.866	1.054 (0.574, 1.932)	0.866
Diabetes (*n*)				
No (278)	103 (37.05%)		Reference	
Yes (15)	8 (53.33%)	0.205	1.042 (0.684, 5.511)	0.212
HBV (*n*)				
No (270)	106 (39.26%)		Reference	
Yes (23)	5 (21.94%)	0.096	0.430 (0.155, 1.192)	0.105
Tumor site (*n*)				
Upper (23)	11 (47.83%)		Reference	
Middle (186)	66 (35.48%)		0.600 (0.251, 1.434)	0.251
Distal (84)	34 (40.48%)	0.436	0.742 (0.294, 1.874)	0.528
cT Stage (*n*)				
cT1 (1)	1 (100.00%)		NA	
cT2 (37)	15 (40.54%)		Reference	
cT3 (196)	73 (38.27%)		0.870 (0.425, 1.784)	0.705
cT4 (59)	22 (37.29%)	0.616	0.872 (0.376, 2.024)	0.750
cN stage (*n*)				
cN0 (40)	23 (57.50%)		Reference	
cN1 (112)	45 (40.18%)		0.496 (0.239, 1.032)	0.061
cN2 (117)	39 (33.33%)		0.370 (0.177, 0.771)	0.008
cN3 (24)	4 (16.67%)	0.006	0.148 (0.043, 0.512)	0.003
cTNM stage (*n*)				
II (53)	25 (47.17%)		Reference	
III (162)	63 (38.89%)		0.713 (0.381, 1.332)	0.288
IV (78)	23 (29.49%)	0.114	0.468 (0.227, 0.968)	0.041
Radiotherapy dose, cGy (*n*)				
≤40Gy (123)	53 (43.09%)		Reference	
>40Gy (170)	58 (34.12%)	0.118	0.684 (0.424, 1.102)	0.119
Chemotherapy regimen (*n*)				
TP (257)	105 (40.86%)		Reference	
Others (36)	6 (16.67%)	0.005	0.290 (0.116, 0.720)	0.008
Duration of nCRT, days (*n*)				
<62 (193)	85 (44.04%)		Reference	
≥62 (100)	26 (26.00%)	0.003	0.446 (0.263, 0.758)	0.003
Time interval (nCRT to surgery), days (*n*)				
0–42 (24)	9 (37.50%)		Reference	
43–56 (52)	15 (28.85%)		0.676 (0.243, 1.876)	0.452
57–70 (89)	30 (33.71%)		0.847 (0.332, 2.161)	0.729
71–84 (53)	22 (41.51%)		1.183 (0.439, 3.185)	0.740
85–98 (31)	15 (48.39%)		1.562 (0.528, 4.628)	0.421
≥99 (44)	20 (45.45%)	0.382	1.389 (0.502, 3.842)	0.527
pre‐nCRT AGR (*n*)				
<1.725 (192)	69 (35.94%)		Reference	
≥1.725 (101)	42 (41.58%)	0.344	1.269 (0.775, 2.078)	0.344
pre‐nCRT NLR (*n*)				
<2.199 (107)	31 (28.97%)		Reference	
≥2.199 (186)	80 (43.01%)	0.017	1.850 (1.113, 3.077)	0.018
pre‐nCRT MLR (*n*)				
<0.321 (149)	50 (33.56%)		Reference	
≥0.321 (144)	61 (42.36%)	0.12	1.455 (0.906, 2.338)	0.121
pre‐nCRT PLR (*n*)				
<99.302 (71)	16 (22.53%)		Reference	
≥99.302 (222)	95 (42.79%)	0.002	2.571 (1.387, 4.765)	0.003
Leukopenia (*n*)				
0 (84)	24 (28.57%)		Reference	
1 (35)	14 (40.00%)		1.667 (0.730, 3.805)	0.225
2 (87)	34 (39.08%)		1.604 (0.846, 3.041)	0.148
3 (67)	28 (41.79%)		1.795 (0.911, 3.536)	0.091
4 (20)	11 (55.00%)	0.189	3.056 (1.124, 8.306)	0.029
Neutropenia (*n*)				
0 (140)	49 (35.00%)		Reference	
1 (23)	8 (34.78%)		0.990 (0.392, 2.500)	0.984
2 (40)	17 (42.50%)		1.373 (0.670, 2.811)	0.386
3 (35)	11 (31.43%)		0.851 (0.385, 1.882)	0.691
4 (55)	26 (47.27%)	0.458	1.665 (0.884, 3.136)	0.114
Hemoglobinopenia (*n*)				
≤1 (257)	95 (36.96%)		Reference	
2 (33)	15 (45.45%)		1.421 (0.684, 2.950)	0.346
3 (3)	1 (33.33%)	0.631	0.897 (0.076, 9.529)	0.853
Thrombocytopenia (*n*)				
0 (193)	71 (36.79%)		Reference	
1 (55)	25 (45.45%)		1.432 (0.781, 2.625)	0.246
2 (36)	12 (33.33%)		0.859 (0.405, 1.823)	0.692
3 (7)	3 (42.86%)		1.289 (0.280, 5.923)	0.744
4 (2)	0 (0.00%)	0.55	NA	NA

Abbreviations: AGR, albumin to globulin ratio; MLR, monocyte to lymphocyte ratio; NLR, neutrophil to lymphocyte ratio; nCRT, neoadjuvant chemoradiotherapy; pCR: pathologic complete response; PLR: platelet to lymphocyte ratio; TP, taxane and platinum agents.

**TABLE 2:** Patient characteristics and univariate analysis for continuous variables associated with pCR.

Continuous variable	Total	Non‐pCR	pCR	*p*‐value	Univariate analysis	
	(*n* = 293)	(*n* = 182)	(*n* = 111)		OR (95% CI)	*p*‐value
Barium_Length, cm	5.231 ± 1.859	5.308 ± 1.823	5.105 ± 1.919	0.365	0.942 (0.828, 1.072)	0.364
pre‐nCRT BMI, kg/m^2^	22.362 ± 2.940	22.246 ± 2.873	22.553 ± 3.051	0.386	1.036 (0.959, 1.123)	0.755
preoperative BMI, kg/m^2^	22.355 ± 3.032	22.114 ± 2.855	22.750 ± 3.277	0.082	1.073 (0.991, 1.161)	0.083
Albumin, g/L	41.553 ± 3.417	41.689 ± 3.336	41.593 ± 3.561	0.816	0.992 (0.926, 1.063)	0.815
Globulin, g/L	26.210 ± 4.198	26.239 ± 4.264	26.161 ± 4.104	0.878	0.996 (0.941, 1.053)	0.878
Hemoglobin, g/L	134.765 ± 15.077	136.523 ± 15.349	131.883 ± 14.221	0.01	0.979 (0.963, 0.995)	0.012
WBC, 10^9/L	6.579 ± 2.007	6.600 ± 1.995	6.543 ± 2.037	0.97	0.986 (0.878, 1.110)	0.812
Neutrophil, 10^9/L	4.278 ± 1.709	4.281 ± 1.766	4.273 ± 1.619	0.966	0.997 (0.868, 1.145)	0.966
Lymphocyte, 10^9/L	1.580 ± 0.521	1.603 ± 0.526	1.543 ± 0.514	0.34	0.800 (0.506, 1.264)	0.339
Monocyte, 10^9/L	0.509 ± 0.172	0.510 ± 0.161	0.509 ± 0.190	0.963	0.963 (0.245, 3.822)	0.963
Eosinophil, 10^9/L	0.130 (0.080, 0.200)	0.120 (0.080, 0.190)	0.150 (0.080, 0.220)	0.192	1.686 (0.455, 6.243)	0.434
Basophil, 10^9/L	0.030 (0.020, 0.040)	0.030 (0.020, 0.050)	0.030 (0.020, 0.040)	0.552	0.044 (0.000, 55.256)	0.392
Platelet, 10^9/L	202.703 ± 70.390	198.934 ± 67.614	208.883 ± 74.621	0.241	1.002 (0.999, 1.005)	0.242

Abbreviations: BMI, body mass index; nCRT, neoadjuvant chemoradiotherapy; pCR, pathologic complete response; WBC, white blood cell.

### 3.2 Analysis of ROC curves

The optimal cutoff values of duration of nCRT, pre‐nCRT AGR, pre‐nCRT NLR, pre‐nCRT MLR, and pre‐nCRT PLR for predicting pCR were 62, 1.725, 2.199, 0.321, and 99.302, respectively. Patients were then divided into low (short) or high (long) groups: short‐duration of nCRT (<62, *n* = 193), long‐duration of nCRT (≥62, *n* = 100); low‐AGR (<1.725, *n* = 192), high‐AGR (≥1.725, *n* = 101); low‐MLR (<0.321, *n* = 149), high‐MLR (≥0.321, *n* = 144); low‐PLR (<99.302, *n* = 71), high‐PLR (≥99.302, *n* = 222) (Table 1).

### 3.3 Univariate and multivariate analysis

Several variables [sex, smoking history, alcohol history, baseline N and TNM stage, chemotherapy regimen, duration of nCRT, preoperative BMI, hemoglobin, pre‐nCRT‐NLR, pre‐nCRT‐PLR, and leukopenia grading] were significantly associated with pCR in the univariate analysis (Table 1 and 2).

Variables were selected for inclusion in multivariate analysis on the basis of their significance in the univariate analysis. After allowance for potential confounders in multivariate analysis, sex, cN stage, chemotherapy regimen, duration of nCRT, pre‐nCRT NLR, and pre‐nCRT PLR retained their statistical significance as independent predictors of pCR. This analysis indicated that female, chemotherapy regimen with TP, short‐duration of nCRT were associated with a higher chance of achieving pCR. The likelihood of reaching pCR decreased with increasing cN stage. Most surprisingly, we found that both high‐pre nCRT‐NLR and high‐pre nCRT‐PLR were associated with the acquisition of pCR. Patients with high‐pre nCRT‐NLR were 1.265 folds more likely to achieve pCR than those with low‐pre nCRT NLR (OR = 2.265, 95% CI: 1.227, 4.181), and those with high‐pre nCRT PLR were 1.284‐folds more likely to achieve pCR than low‐pre nCRT PLR counterparts (OR = 2.284, 95% CI: 1.147, 4.548) (Table 3). Furthermore, we found patients with pre‐nCRT NLR ≥ 2.199 showed a significantly higher pCR rate than those with lower value (43.011% vs. 28.972%, *p* = 0.017). The pCR rate was also higher in patients with pre‐nCRT PLR ≥ 99.302 (42.793% vs. 22.535%, *p* = 0.002) (Table 1).

**TABLE 3:** Multivariate analysis for variables associated with pCR.

Variable	Multivariate analysis	
	OR (95% CI)	*p*‐value
Sex (female vs. male)	0.297 (0.143, 0.620)	0.001
cN stage		
cN1 versus cN0	0.654 (0.291, 1.467)	0.303
cN2 versus cN0	0.403 (0.178, 0.911)	0.029
cN3 versus cN0	0.234 (0.063, 0.872)	0.03
Chemotherapy Regimen (TP vs. others)	0.230 (0.081, 0.647)	0.005
Duration of nCRT (<62d vs. ≥62d)	0.388 (0.217, 0.693)	0.001
pre‐nCRT NLR (<2.199 vs. ≥2.199)	2.265 (1.227, 4.181)	0.009
pre‐nCRT PLR (<99.302 vs. ≥99.302)	2.284 (1.147, 4.548)	0.019

Abbreviations: NLR, neutrophil to lymphocyte ratio; pCR, pathologic complete response; PLR, platelet to lymphocyte ratio; TP, taxane and platinum agents.

### 3.4 Nomogram

**FIGURE 2:**
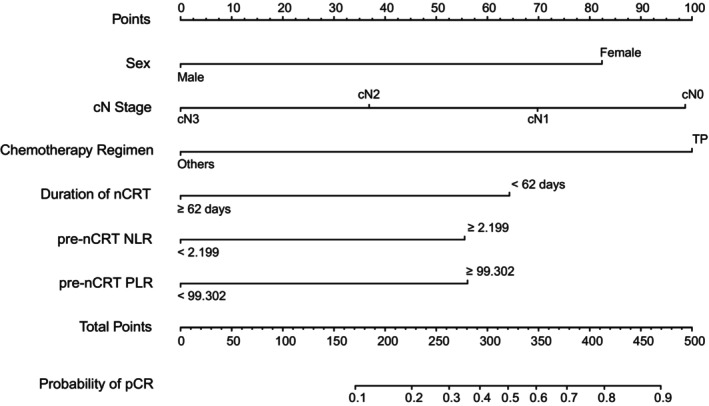
Nomogram for prediction of pCR after nCRT in patients with ESCC. For each patient, seven variables are assigned points on a nomogram, represented by seven lines moving upward. The sum of these points is then located on the “Total Points” axis. A line is drawn downward from this point to predict the probability of achieving pCR. ESCC, esophageal squamous cell carcinoma; pCR, pathologic complete response; PLR, platelet to lymphocyte ratio; nCRT, neoadjuvant chemoradiotherapy; NLR, neutrophil to lymphocyte ratio; TP, taxane and platinum agents.

**FIGURE 3:**
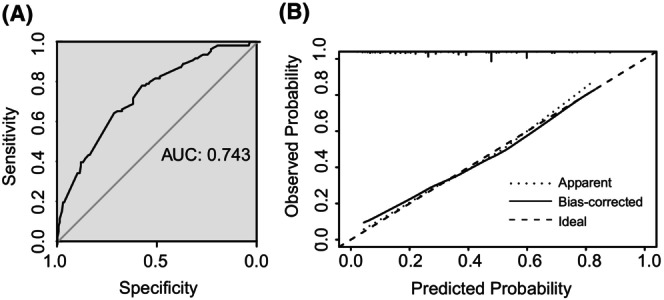
Evaluation of the performance of the nomogram by discrimination and calibration. (A) ROC curve of the nomogram. (B) Calibration plots of the nomograms.

A nomogram was developed to predict pCR on the basis of multivariate logistic regression coefficients. The nomogram demonstrated that combining six variables can increase the probability of predicting pCR to as high as 90% if a patient score >470 points (Figure 2). The C‐index of the nomogram was 0.743 (95% CI: 0.686, 0.800), indicating that the model has a high accuracy in predicting. The calibration curve displayed acceptable agreement between the prediction and actual observation, confirmed by the Hosmer–Lemeshow goodness‐of‐fit test, with *χ* ^2^ = 1.021, 2 degrees of freedom, and a *p* value of 0.600 (>0.05) (Figure 3).

## 4 DISCUSSION

Esophagectomy does not provide clinical benefits to patients with advanced ESCC who achieve pCR with nCRT, and there is currently no reliable model for predicting pCR that is widely used in clinical practice. Therefore, there is a need to develop a new predictive model. In this analysis, the percentage of patients with pCR was 37.88%. By combining variables that were independently associated with pCR and internal validation by bootstrapping, we developed a nomogram (incorporating six different variables) that showed a high accuracy and consistency for predicting pCR in ESCC patients. We found that the cN stage and chemotherapy regimen had high contribution in the nomogram followed by sex and duration of nCRT. Surprisingly, different from the previous studies, we found that high pre‐nCRT NLR and high pre‐nCRT PLR were associated with pCR.

Researches have indicated that early‐stage tumors are more likely to achieve pCR after nCRT compared to advanced tumors, possibly due to their lower tumor burden.^8^, ^25^ Similar to our study, Nusrath S. et al also found that pretreatment radiographic node‐negative status (cN0) was associated with pCR after studying 321 patients with esophageal cancer.^25^ Previous studies also suggested that cT stage were correlated with pCR, potentially due to the use of endoscopic ultrasound to obtain more precise staging information.^8^ However, our staging of tumors relied on the CT findings before nCRT, which highly relied on interpretation by radiologists and oncologists. To overcome this limitation, recent researches have explored the potential of deep learning techniques for CT analysis, which could enhance the accuracy of predicting pCR.^26^, ^27^

That female gender leading to a higher probability of pCR was consistent with other studies, suggesting that sex hormone patterns may have a role in women's superior ability to cope with cancer.^8^, ^14^ The current study also indicated that the chemotherapy regimen of TP was significantly correlated with a higher pCR rate compared with the other category. The other classification either did not use a combination regimen of TP, or the dose of taxane or platinum was insufficient. A meta‐analysis of clinical studies confirmed that the benefit (including but not limited to pCR) of taxane‐based therapies over PF in all types of therapy in patients with ESCC.^28^ Duration of chemoradiotherapy was another indicator. Limited by PS scores and hematologic toxicity of the patients, the selection of chemotherapy regimens and cycles, and accessibility of medical resources during nCRT, the duration of chemotherapy could be prolonged. It is easy to comprehend that the effectiveness of nCRT was considerably diminished by prolonging the treatment duration, particularly in terms of the efficacy of radiotherapy.^29^

This article examined the interval between the completion of nCRT and surgical resection. The categories of time interval (nCRT to surgery) were based on a study published in JAMA Surgery: 0–42 days, 43–56 days, 57–70 days, 71–84 days, 85– 98 days, and 99 or more days.^23^ The first two boundaries, 42 and 56 days, were set based on the existing studies suggesting waiting time should be around 6–8 weeks. Additional intervals of 14 days were selected to establish boundaries up to 99 days. A meta‐analysis showed that increasing the time interval from nCRT to esophagectomy was associated with significantly higher pCR rates in esophageal adenocarcinoma, but delayed surgery did not result in a higher pCR rate in ESCC.^30^ Our findings were consistent with previous studies.

To avoid the effect of non‐chemoradiotherapy medications on blood counts and the convenience of indicator acquisition, we did not explore hematological indicators during and after the end of nCRT, except for hematotoxicity. Specifically, our results indicated that a high pre‐nCRT NLR and PLR correlated with a higher pCR rate, which contradicted previous findings and highlighted the contentious nature between inflammatory markers and pCR. Earlier researches indicated that either a low pre‐nCRT NLR and PLR may enhance the pCR rate or have no impact at all. These studies had the following characteristics. First, the sample sizes of these studies were small. For example, two studies that said that pre‐nCRT NLR and PLR were not related to pCR had sample sizes of 84 and 101 ESCC patients, respectively.^15^, ^31^ Second, the confounding factors were not excluded by multivariate analysis. A study involving 365 patients with ESCC reported that low pre‐nCRT PLR levels were linked to achieving pCR, whereas there was no link between pre‐nCRT NLR and MLR and pCR. Nonetheless, the study did not conduct a multivariate analysis.^16^ Third, the time span covered by the inclusion of patients was quite extensive, during which the collection of clinical data and the examination of hematological indicators may be subject to errors and variabilities. A study reported that patients with pre‐nCRT NLR levels >2.1 had a lower likelihood of achieving pCR compared to those with lower levels, but a total of 306 patients were enrolled over a period of 14 years, from 2003 to 2017.^14^ Last but not least, the previous chemotherapy regimen in nCRT was basically a PF regimen, but the vast majority of our patients were treated with a taxane‐based regimen, which will be discussed in detail in the following paragraph.^14^, ^17^

We summarized the following factors that may possibly explain our findings, which showed that a high pre‐nCRT NLR and PLR were correlated with a higher pCR rate. Although tumor‐infiltrating lymphocytes (TILs) have multiple components, high absolute lymphocyte count nadir during nCRT was considered with a higher pCR rate.^14^ The amounts of neutrophils and platelets were critical in determining the magnitude of NLR and PLR. Our initial focus was on neutrophils. Even though the majority of researches indicated that neutrophils play a pro‐tumor role, they actually have the ability to exert both tumor‐enhancing and tumor‐inhibiting effects due to the heterogeneity of neutrophils. Neutrophils play a crucial role in anti‐tumor responses by directly killing tumor cells and participating in cellular networks that mediate antitumor resistance.^32^ In most cases, high neutrophil infiltration was linked to a poorer response to chemotherapy and radiotherapy. However, there were some notable exceptions such as colorectal cancer, gastric cancer, and ovarian cancer, where increased levels of tumor‐associated neutrophils were associated with a better response to chemotherapy.^33^ The role of neutrophils in esophageal cancer deserves further exploration. In contrast to neutrophils, platelets have been recognized as an active player throughout tumorigenesis, including tumor growth, tumor cell extravasation, metastasis, and drug resistance. Additionally, thrombocytosis in cancer patients is associated with adverse patient survival.^34^ Luckily, the use of chemotherapeutic agent paclitaxel has been proved to inhibit platelet−tumor cell interactions, thereby suppressing the process of tumor epithelial−mesenchymal transition, reducing distant metastasis, and reversing tumor immunosuppresion.^35^

The next focus was the roles of the taxane‐based regimen. Taxanes are a type of cytotoxic chemotherapy drug that target microtubules and are used to treat a variety of solid tumors. Indeed, taxanes have a multifaceted mechanism of action that affects cellular oncogenic processes such as mitosis, angiogenesis, apoptosis, inflammatory response, and ROS production. Among these processes, the inflammatory response is especially noteworthy.^36^ Taxanes can induce micronucleation, leading to cGAS/STING signaling, which activates innate immunity and causes inflammation. Taxanes can also stimulate cancer cells to produce IFN‐β and increase the presence of immune cells within tumors. As a result, taxanes directly promote an anticancer immune response by stimulating macrophages to kill cancer cells and indirectly by causing the secretion of proinflammatory cytokines that activate dendritic cells, natural killer cells, and tumor‐specific cytotoxic T‐lymphocytes. Furthermore, taxanes is able to modulate myeloid‐derived suppressor cells and eliminate Tregs. In short, taxanes can improve the tumor microenvironment in a comprehensive manner.^36^, ^37^

Other nomograms have also been developed to predict pCR. Initially, nomograms were constructed using basic information on patient and tumor characteristics. For example, Toxopeus EL et al found that female sex, squamous cell histology, poor differentiation grade, and low cT stage were predictive for a pCR, but with a c‐statistic of 0.64.^8^ With incorporating hematological biomarkers into the predicting model, the accuracy of the prediction has been elevated to 0.73–0.75.^14^, ^38^ To improve the accuracy of prediction, other factors began to be included in the nomogram. Molecular biomarkers and functional imaging are the main direction. Genes, serum, and pathology specimens can be utilized as sources for molecular biomarkers. A four‐gene‐based immune signature (SERPINE1, MMP12, PLAUR, and EPS8) was built based on the verified differentially expressed immune‐related genes achieved a high accuracy with an AUROC of 0.970.^39^ A significant association was observed between pCR and lower serum levels of four metabolites–arabitol, glycine, l‐serine, and l‐arginine, as well as EGFR overexpression determined by immunohistochemistry.^40^, ^41^ Evaluating changes in parameters of both 18F‐fluorodeoxyglucose PET and diffusion‐weighted magnetic resonance imaging could provide complementary value for assessing pathological response after nCRT.^42^, ^43^ With the rapid development of deep learning, it is now possible to use routine CT images to predict pCR with good accuracy.^26^, ^27^ Despite not utilizing the above indicators in our study, our model may apply to the majority of patients in clinical settings, as these indicators and methods of analysis are not routine clinical practice.

There are some drawbacks and limitations in this study. Limitations of this study included its retrospective design and its focus on a single center. Besides, based on the cohort size of 293 patients and pCR rate of 37.88%, a maximum of 11 predictive parameters was allowed to be selected for response prediction. After conducting the univariate analysis, we identified 12 factors that were subjected to further analysis. In addition, the impact of pCR on outcome was not evaluated in this study. Finally, although we utilized internal validation through bootstrapping, it is important to conduct external validation on an independent cohort of patients prior to recommending the clinical application of the nomogram presented in our study. However, we believed that the results were reliable due to the following reasons: this study was conducted in a large cohort of patients, the independent variables underwent screening before conducting the multivariate analysis, the OR values, and 95% CI of the results were relatively standard, internal validation has been performed on the nomogram, the Hosmer–Lemeshow goodness‐of‐fit test of the model demonstrated the success of the nomogram, and the findings of the indicator features were reasonably comparable to those of studies of similar nature.

## 5 CONCLUSION

In this study, we demonstrated that sex, cN stage, chemotherapy regimen, duration of nCRT, pre‐nCRT NLR, and pre‐nCRT PLR were significant predictors for pCR in ESCC patients after nCRT. Furthermore, a nomogram incorporating these biomarkers has been constructed with high accuracy and consistency. To our knowledge, the present study was the first attempt to establish a predictive model for pCR when taxane‐based therapy as the primary chemotherapeutic approach for ESCC by collecting easily obtainable clinicopathological data and hematological markers. This model could be helpful for patients and clinicians in making clinical decisions.

## AUTHOR CONTRIBUTIONS

**Guihong Liu:** Conceptualization (lead); data curation (equal); formal analysis (equal); investigation (equal); methodology (equal); project administration (equal); resources (equal); software (equal); supervision (equal); validation (equal); visualization (equal); writing – original draft (equal); writing – review and editing (equal). **Tao Chen:** Data curation (equal); formal analysis (equal); investigation (equal); methodology (equal); software (equal); writing – review and editing (equal). **Xin Zhang:** Data curation (equal); investigation (equal); resources (equal); software (equal). **Binbin Hu:** Data curation (equal); investigation (equal); methodology (equal); software (equal). **jiayun Yu:** Data curation (equal); formal analysis (equal); investigation (equal); methodology (equal); supervision (equal); visualization (equal); writing – review and editing (equal).

## FUNDING INFORMATION

This research was funded by National Natural Science Foundation of China, grant number: 82102897.

## CONFLICT OF INTEREST STATEMENT

The authors declare no conflict of interest.

## ETHICS STATEMENT

The study was carried out in compliance with the principles outlined in the Declaration of Helsinki and received approval from the Institutional Review Board of West China Hospital (2023‐1230). Informed Consent Statement: Patient consent was waived due to the retrospective nature and non‐interventional aspect of the study as determined by the ethical committee of West China Hospital.

## Data Availability

The raw data supporting the conclusions of this article will be made available by the authors, without undue reservation.
